# Landed property as collateral to access credit for housing development in Ghana: The case of Northern Region of Ghana

**DOI:** 10.1016/j.heliyon.2023.e17646

**Published:** 2023-06-26

**Authors:** David Asante Edwin, Evam Kofi Glover, Edinam K. Glover

**Affiliations:** a Faculty of Social Sciences, University of Helsinki, FI- 00014 Helsinki, Finland; b School of Allied Health Sciences, University for Development Studies, Tamale Campus, Box TL 1883 Tamale, Ghana; c Circular Family in Bangladesh (CFB), Natural Resources Governance, Environmental Law & Policy, Liaison Office, Hatirpool, Dhakar, Bangladesh

**Keywords:** Collective property, Credit officers, Financial institutions, Ghana, Housing development, Landed property

## Abstract

***Property in the commons, or the Washington Consensus, as it is known in other circles***, posits that landed property and access to formal credit are directly related. Whether landed property improves access to formal credit or not has been at the centre of the debate, with varying practical evidence, especially for the Global South. Another related dimension of the debate concerns the implications of family-help-mortgage arrangements using intra-family transfer of land as collateral to support a member's mortgage by placing a charge on the collective property. This paper investigates the use of landed property as collateral to access formal credit from financial institutions for housing development in the Tamale Metropolitan Area (TMA) in the Northern Region of Ghana. The primary research question is: ***does*** formal documentation of landed property as collateral help in accessing formal credit for housing development in TMA? The study involves the use of in-depth interviews to investigate the opinions of estate developers, credit officers of universal banks, and land title holders on the relative importance of landed property and access to credit for housing development. The data are analysed by means of thematic content analysis. The findings indicate that even though landed property may contribute to the decision to grant formal credit, it is not a turn-key solution easily accepted in the final lending decision. This study adds to the body of knowledge primarily by evaluating the effects of reforms to increase credit access with landed property as collateral in Ghana, investigating the implications of family-help mortgage arrangements, including the use of intra-family assets as collateral in obtaining loans for investment at the micro-level in Ghana. The paper concludes that while formal documentation of land rights matters in the broad scheme of estate financing, it is not sufficient to bring about more immediate access to credit, especially for those who lack regular or reliable systems of income that could be critical in ensuring the payback of loans contracted using landed property as collateral for housing development.

## 1 Introduction

One of the most significant impacts of urbanisation is on urban housing and land-use management. In the Global South, mostly in Africa and Asia, the rate of urbanisation has been increasing steadily [1,2]. Southern societies increasingly face the challenge of, on the one hand, implementing urban development in a way that accommodates the expanding urban populace and meets its basic service needs, and on the other, reducing the associated negative effects of environmental degradation, high resource consumption, pollution, and social and economic exclusion [3]. According to Ref. [4], besides job insecurity in the larger cities of African countries, the challenge of a housing deficit and translating urban development policies that truly achieve the challenge of meeting the growing need for housing and services is a huge concern.

Research suggests that only 15% of urban dwellers in Africa have access to housing finance, while the remaining 85% are left without [5]. In the face of the ever-rising housing demand and increasing housing value in the bid to fill-in the deficit of housing needs, conventional housing finance systems in Africa have been found wanting. They have hardly been able to meet the challenges brought by this social change (ibid.).

The study focuses on deficits in housing supply in many urban areas in Ghana as a point of departure, and demonstrates that the challenge of filling-in the deficit in housing needs in urban Ghana has increasingly become a public concern. The literature [6,7], suggests that the shortage of housing continues to be one of the most critical socioeconomic challenges facing Ghana as a country. It is estimated that Ghana has a housing deficit of 2 million units and therefore it is critical for a minimum of almost 200,000 housing units to be built annually. In 2012, it was estimated that Ghana faced a housing deficit of well over one million houses [6,8]. The observation of [9] is that the situation is compounding annually due to the government's perennial inability to develop effective and sustainable housing financing schemes to meet the housing needs of the people of Ghana.

To overcome this challenge, the literature suggests that a sustainable housing financing system is critical [[10], [11], [12]]. Such a facility is even more demanding in Global South countries like Ghana, where emigration into cities has overwhelmed housing facilities, resulting in sprawling slums [13,14]. Even though accessible credit opportunities have generally been hailed as a driving force in ensuring sustainable housing schemes, various reasons for the limited flow of bank credit to households and firms has been a theme in numerous research studies in these countries. In Ghana the challenge has been duly recognised. Aryeetey [15] observed the discrepancy between the reluctance of financial institutions to make credit available because households and enterprises largely lack adequate acceptable collateral to support their loans. Available statistics show that, in Ghana, an estimated 79% of micro and 83% of small-scale enterprises are credit constrained, compared with 62% and 68% respectively in Malawi (ibid.:164). Consequently, in an environment where prospective investors lack collateral to support credit to invest in housing schemes, personal savings, selling of assets, and remittances from relatives abroad have become the main-stay of financing for private housing construction and purchases [12,16]). Due to the inadequate funds raised from these sources, in most cases it takes over a decade to complete the construction of one property [12,17].

Fleisig et al. [18], were of the view that, since a significant proportion of the working population in Africa are employed in the informal sector, application for bank credit becomes daunting because of the lack of acceptable collateral and the perceived unreasonable collateral requirements by the Banks, which many in this sector cannot afford. The situation is commonly held to represent a major barrier to real estate development or housing delivery in many African countries. To offset this barrier, the story of Ghana suggests that, prior to the mid-1980s, the strategy of the Ghanaian government was to serve as the principal supplier of housing [[19], [20], [21]].

The housing programme was aimed at replacing informal houses such as shanty towns and ghettos, which had resulted from increasing population growth and urbanisation. Unfortunately, the subsequent economic recession and the implementation of the Structural Adjustment Programme forced the State to abandon this perspective and assume what was termed the ‘enabling role’. The ‘enabling role’ of Government meant that the State primarily acted only to facilitate and enable the private sector to take the centre stage in housing delivery. Consequently, two major strategies were enforced. In the first, the State created financial institutions to provide credit facilities to empower people, and in the second, favourable conditions boosted the private sector through financial and tax incentives to enhance the role of private real estate developers in increasing provision of affordable houses.

The records [[21], [22], [23]] suggest, however, that these interventions in housing delivery could not effectively meet the goal of solving the housing deficit, especially among urban low-income earners. The literature posits that housing finance is the critical factor in housing delivery [21,24]and the fashion in which this scarce resource is made available to property developers is of considerable significance in ensuring healthy housing delivery [21,25].

For [12,26], the housing deficit, especially among urban low-income earners in Ghana, is also a factor in the poor mortgage market. The mortgage market remains largely underdeveloped, with major challenges like short terms of mortgage and high interest rates, with down-payment requirements sometimes making up half of the total cost of the residential facility. Given this situation, it is prohibitively expensive for most people, especially lower and many middle-income households, to participate in the mortgage market.

A land-based approach to financialisation of housing has emerged strongly in the literature over the years as a source of financing to fill-in the deficit in housing in the Global South [26]. In this context, the United Nations Centre for Human Settlements (UNCHS 1999 cited in Ref. [28]), has recognised security of land tenure and better governance as two necessary incentives for investment in land and housing. A key justification for secure property rights to land is therefore that such rights promote collateral for bank loans [9,[29], [30], [31], [32], [33]], which facilitates innovative housing financialisation. In the same vein [26], has pointed out that tenure security plays a central role in facilitating urban residents’ own investment and development in the housing sector. It has been observed, however, that besides land security, accessible and inexpensive housing finance is a key restriction on reasonable housing in Africa.

Against this background, several scholars have considered a critical shift towards a more comprehensive conceptualisation of security of tenure as an incentive for investment in land and housing. A key justification for secure property rights to land is therefore that such rights promote collateral for bank loans in housing financialisation.

As indicated by Ref. [34], land tenure security emerges from the concurrent presence of three factors, namely: *“legal security,* de facto *security and perceived tenure security*.” These three elements together are seen as critical for a reasonable meaning of one's right or interest in the land. Domeher et al. ([35]:163), explains that security of land tenure includes two types of validation or approval – “*state validation by legal recognition and social acceptance at the local level*”. To Domeher et al. these two types of validation should be available to fortify tenure security. Without approval at the local level, legal recognition alone may not prevail with regards to making legitimate claims, while local recognition alone might be debilitated without the support of the national legal framework. Incorporating local values on property ownership into the national legal framework will aid in accomplishing the two types of recognition (ibid.).

The work of [36] lends support to this school of thought. Among other strategies [36],discusses how the Government of Turkey employed housing financialisation through new legislation; creating financial frameworks that enhanced speculation by domestic and international capital on land and housing as assets; enclosing public land and exploiting informal types of tenure; making assets of land and housing by developing revenue-sharing urban regeneration projects; and using coercive legal and penal force to outlaw informal development, and to suppress resistance to state-led development efforts.

In this sense [36], demonstrates how a government could strategically enforce policy to promote the regulatory, legislative, and financial conditions necessary to ensure legal security, de facto security, and perceived tenure security bundled in concert as formidable financial capital to penetrate land and property markets. This is anchored on the fact that any unjustifiable attempt to curtail one's security of tenure can trigger enforcement action from the state or other designated authority [35,37].

In this regard [28], observes that the main thought behind land title registration in the Global South, including Ghana, is to safeguard the security and certainty of land rights in customary land tenure. People have the general fear that customary land tenure systems or traditional landholding institutions do not provide adequately secure and certain access to land, which is critical for investing in land. In this respect, formal title registration in customary land tenure systems is seen as critical for the security and certainty of property rights to land. With this clarity, agencies, including donors from western countries and international development organisations such as the International Monetary Fund (10.13039/100003887IMF) and the 10.13039/100004421World Bank, can continue to push for property owners to use their properties as collateral for credit [9,[29], [30], [31], [32], [33],^38^]. This perspective therefore holds that property registration could improve the collateral properties of land through its security enhancing ability; making it a better, more secure, and acceptable form of collateral that can be used to secure investment credit [29,33]. By acquiring secure property titles, property owners who do not have funds to invest can thus obtain credit to undertake their desired investment activities through the collateralisation effect.

In order to ease the stress that Ghanaians are subjected to in accessing loans, the World Bank, in collaboration with the International Finance Cooperation (IFC) and the Bank of Ghana, launched a collateral registry in Ghana in April 2018. The collateral registry is a body established by Parliament under the Borrowers and Lenders Act, 2008 [39] to secure credit facilities provided by lenders. The decree of the Collateral Registry is a response to the teething problems in Ghana's credit market. The issue of inadequate credit eventually constrains the flow of credit to Small and Medium-sized Enterprises [SMEs] in particular, as well as to households and the general economy [39,40].

To offset this challenge, several scholars have considered a critical shift towards a more comprehensive conceptualisation of security of tenure that addresses the legal, economic and social aspects of landholding [34,41]. If we consider the argument as posited by some scholars [[4], [34], [42], [44]], then land tenure security is as good as any collateral for legitimate property owners to access credit from banks.

The examination of the relationship between housing and financial sector became popular during the 2008 global financial crisis [43]. But prior to this global crisis, scholars such as [44], observed that land could function as a financial asset besides its traditional role as a factor of production or consumption good. Further, she noted that a theory that explains the relationship between landowners’ power and finance, as well as the relationship between real estate and the financial sector is pertinent [27].

A review of the literature suggests that the relationship between real estate and the financial sector has been explored in a number of countries in the Global South [35,37,[45], [46], [47], [48], [49]]. Indeed, in Ghana, a number of these studies explored various aspects of the challenge [21,25,45], including constraints on real estate finance, the development of the mortgage market, and the importance of secure titles in the lending process. Admittedly, even though these studies have helped examine the link between land registration and access to credit, they have largely been focused on either the demand or the supply side of the mortgage market. Except for some very few [35,37,46,47], studies evaluating the impact of reforms to enhance credit access with landed property as collateral are less common in Ghana.

What seems missing in previous studies has been the micro-level, implications of family-help mortgage arrangements, including using intra-family asset as collateral in obtaining loans for investment. Indeed, in customary practice, family-help mortgage arrangement is a strategy that has been used for ages in traditional circles in Ghana during times of individual or family need. Even with the promulgation of the Intestate Succession Law, 1985 (PNDCL^1^ 111) [50], experience suggests that Ghanaian families may not follow the provisions of the Intestate Law in dealing with the self-acquired property of a deceased father, fearing it would dissipate their property [51]. Some families prefer keeping the property in a pool for the whole family, especially in the case of land. This is particularly the case when the piece of property bequeathed is relatively small and cannot easily be shared except by selling the land and distributing the proceeds. However, distributing such property is a challenge in northern Ghana, where Total Fertility Rate is high, and polygyny is the norm [52]. Polygynous units with different uterine families mean a high dependency ratio. The prospect that fragmentation of the property in such circumstances could devalue its economic significance prompts families to keep such property in the pool. The advent of formal banking reinforced the customary use of such assets.

The case study of (TMA) offers, among other things, an opportunity to investigate this social current in all its facets (monogamous family units as well as uterine families of polygynous homes) as a common phenomenon. It is significant to investigate, among other things, how actors within Dagbon family structures fare in using this revolving titled land-based credit-accessibility instrument, especially within polygynous homes based on uterine-families as the unit. In economic literature therefore, where such an asset has been dully registered and property rights secured, it is considered an advantage for economic development and the efficient use of resources [53]. It serves as a strategy that supports individual buyers with low deposits to obtain loans from banks, using the collective property as security for their mortgages.

The strategic importance of a collective family asset being pledged as security for repayment of a loan is therefore not new to the traditional system in Ghana. To date, however, there has been no empirical study in northern Ghana to investigate the dynamics and implications of such intra-family properties in the assets-based credit-accessibility system. Previous studies [25,28,47] have largely explored only the implications of individuals applying for credit with private titled property. This study therefore contributes to the literature, by interrogating the modalities by which the widespread use of shared titled-nuclear-family-assets as a traditional arrangement fits into the modern banking system, and its implications for the family as a unit.

The current study also differs by exceptionally analysing both the demand and supply side of the mortgage market. Among other things, the study evaluates the opinion of both lenders (universal banks) and borrowers (title holders, developers) on the prerequisites for credit access with landed property.

Another contribution of this study relates to the implication of land management in the context of ethnic diversity in Ghana. The situation means that different ethnic groups continue to reference different forms of succession tenets applied side by side to the formal. Given this background, it is obvious from the literature that even though work has been done on the prospects of secure property rights to land and its implications in Ghana [21,25,28,35,47], the challenge remains that previous studies tended to lump different ethnic groups together for analysis. In such analysis, the critical peculiarities that inform access to housing finance with landed property in the multi-ethnic environment of Ghana are inadvertently glossed over. The literature suggests that customary practices in northern Ghana, with its diverse ethnic groups, make it relatively difficult to generalise findings to the whole population. What is critical here is for small scale studies to attempt to capture ethnic implications for secure property rights to land and how this is used as capital. The current study therefore seeks to explore the meaning of land tenure security in Tamale (the regional capital of the Northern Region and among the Dagomba) as collateral for Bank loans in housing financialisation.

This study seeks to answer the main question: does formal documentation of landed property as collateral help in accessing formal credit for housing development in the Tamale Metropolitan Area in the Northern Region of Ghana? The findings of this study could be useful to the government, financial institutions, property developers, and non-governmental organisations seeking to design an innovative and sustainable housing finance system in Ghana.

## 2 Theoretical perspective: basic principles of property and tenure theories

This paper attempts to interrogate the implications of land title registration for land security as collateral in seeking Bank loans in housing financialisation in the ever-sprawling city of Tamale. This has implications for gentrification, rising housing costs, and rising housing value in the bid to fill-in the deficit of housing in the city. A land-based approach to financialisation has been noted generally in the literature as a source of financing [21,25,27,28,35,47]. The theoretical framework that guides this study is therefore based on property theories that support the importance of landed property as collateral to access credit for housing development. In much of the Western world, the economic view is that land is simply a factor of production and therefore assumed to conform to factor market characteristics.

Following this perspective, the [54] and, later [55], analyses of property are foundational to market theories in their support for the individualisation of property within ethical limits [56]. These theories preceded those arguing the natural inevitability of individualisation as well as those on the economic advisability of individualisation. These are, however, opposed to common property theorists who do not see individualisation as a catalyst for development.

Even though Locke is thought of as supporting the individualisation of landed property, which is part of neoclassical economic theory, the difference is that the neoclassicist is not constrained by morality but must obey the laws of economic self-interest in order to support the economic system. Locke, however, held that the prevailing morality of the “rule of propriety” is paramount in preventing inequity in the world [57]. Theorists such as [58] doubted the ability of ethics to provide a workable solution to problems of finite resources. They held the view that, within the considerations of the open access system, ethical behaviour would doom the highly moral individual to a disadvantaged position. They believe that this would lead to their exploitation and ultimately to their elimination from the user community.

For this paper, we take our theoretical point of departure from the series of arguments made by Hernando de Soto in his books - *The Other Path* [59]*; and The Mystery of Capital: Why Capitalism Triumphs in the West and Fails Everywhere Else* [29]. He argues that formal property rights are essential to economic growth and poverty alleviation by exposing the capital potential of properties held informally by the poor (especially in the Global South). He pointed out that capital formation and economic growth are dependent on access to credit and investment in the future. In addition, de Soto argues that, to achieve secure property rights, the society must incorporate the traditional informal, unwritten rights into a written, formal, legal property rights system. In this respect, an integrated system of standard legal titles becomes critical. De Soto underscores the codification of informal property rights into writing in a legal system of property titles as the way to securing property rights. To him, land titling is a critical mechanism through which property rights can be achieved.

De Soto asserts that the key to stimulating economic growth in the Global South lies in codifying informal property rights within a written formal legal system. The pledging of landed property as collateral to serve loans is a common, important part of the credit acquisition process. Indeed, personal collateral and commitments is a common feature of many small business credit contracts. For analysts like [29] therefore, land commodification assumes that land and land-related rights can be individuated from traditional notions of communal ownership into land secured by rights of the individual that could be traded and transformed into a capital mobilisation instrument [60].

Conventional theorising has largely followed this line of argument. UNCHS (1999, cited in Ref. [28]) recognised security of land tenure and better governance as two main necessities that require immediate and urgent attention. A key justification for secure property rights to land is that such rights provide incentives for investment in land and sustainable development.

The literature [[61], [62], [63]] has also argued that the lack of formal title registration in customary land tenure systems creates a sense of insecurity and uncertainty of property rights to land. However, other theorists argue that a private, exclusionary model that transforms land into a commodity may bring more challenges without due consideration of social, ethical, and cultural concerns, which are invariably much better addressed by the traditional communal ownership scheme [[64], [65], [66], [67], [68]]. Opponents of the exclusionary model have also pointed out that titling schemes and privatisation of community-owned land remains a challenge within broader political economic considerations. The work of Obeng-Odoom [69,70]) in a critique of de Soto's theorisation about land, concluded that rather than improving the lot of the poor, such a theory is grossly inadequate for solving the problem and would inevitably serve to increase poverty instead.

Against this background, the following section attempts to analyse the commodification of land development rights in Africa and also explores the scholarship of commodification of land in Ghana.

## 3 Commodification of land in africa and existing scholarship on Ghanaian land markets

Drawing on the work of scholars like Joseph Schumpeter, Karl Polanyi, and Henry George, Obeng-Odoom demonstrates that de Soto's thesis is flawed in many respects and has little potential to facilitate investment that can generate capital accumulation, especially in Africa [69,70]. The two opposing schools of thought demonstrate some of the challenges Ghana faces in its quest to enhance general social development through legislation for regulating the use of land specifically. The latent function of the transformation process is the emergence of ambiguities that embroil every facet of the social structure. To address these hydra-headed challenges, the Ghana Land Policy document, for example, has about ninety different pieces of legislation attached to the legislative framework regulating the use of land in Ghana [71,72]. Before the promulgation of the Intestate Succession Law 1985 (PNDCL111) [50] and The Head of Family Accountability Law 1985 (PNDCL 114) [73], the family ‘inherited’ a deceased person who died intestate. The PNDCL 111 only seeks to intervene specifically in the inheritance of the personal property of a person who dies intestate. In this sense, the law does not intervene in lineage, extended family property, in stool land or corporate tenure landholding arrangements under the customary tenure system.

Much as these legislations have brought great relief to the small family unit, they have not completely removed the challenges that emanate from intrafamily concerns regarding the right of any one member of the said nuclear family unit to use that common property as collateral. Despite the gradual shift towards appreciation of titling schemes and privatisation of community-owned land in Ghana, little research has so far been done on situations where family registered assets are sought after as collateral to back the loan applications of individual family members.

Among other things, this paper also seeks to draw attention to such grass-roots issues and negotiations over cooperative property within the small family network. For the purposes of this paper, we define cooperative property as property that jointly belongs to more than one individual within the small family unit (be they members of the various uterine-families within a polygynous family or members of a nuclear family) by courtesy of common inheritance from a parent. As a common-family-asset (commonwealth) across uterine-families in a polygynous union, for example, such cooperative property may be controlled in distinct ways. Even though in theory every individual family member has a stake in this property, it is expected that the oldest male sibling within the family is granted the right to become the main representative holding the property documents. In this sense, there is a legal relationship between the members of that family and the state enforcing a possessory interest or legal title in that property. The respective party's discretion with regard to the use of that property is to be clearly defined, and the parties involved might expect their wills to be unanimous in how the property could be used. It is therefore expected that when no opportunity for dispute with any member exists, then the use to which the commonwealth is applied is to be reached only by consensus, where their own will as regards the use of the property is regarded as sufficient and absolute.

Even though such a commonwealth has featured prominently as collateral for members as individuals seeking loans, there has to date been no rigorous study on how this is negotiated within the small family unit, including within the polygynous union specifically. Clearly, there is a dearth of research on the decision-making process within the small family unit that leads to the temporal transfer of family documents to one member for personal use as collateral and what happens when this person is unable to pay back. As its contribution to knowledge, this work seeks, among other things, to look critically at the rights of each individual member of the family to the family property and what negotiations become necessary in transferring these rights to one member who needs collateral for loans.

It is also clear from the literature reviewed that, even though a lot of scholarly work has been done on the subject of land as collateral, there is hardly any work specifically looking into intrafamily small units (including polygynous families with uterine units) and how the use of the commonwealth is negotiated in support of individuals seeking to use this family property as collateral for loans. Among other considerations, the purpose of this study therefore is to explore the intricacies in the negotiation of the commonwealth as collateral for individuals, how this is conveyed, and what happens in case of default of loan payment and what is done to retrieve the said property.

Two legislative interventions relating to property succession and landholding accountability on access to land by individual family members are critical for our analysis. These interventions include the Intestate Succession Law 1985 (PNDCL 111) [50] and The Head of Family Accountability Law 1985 (PNDCL 114) [73], which have potential relevance to land access, including even at the level of the smallest family unit. In this arrangement, ownership of the property reverts from individual ownership (of the deceased) to land vested in the nuclear family as a corporate unit [74]. noted that no individual can claim sole ownership to such a land. It is however argued that when the family functions as a collaborative team, the family unit and the land held by the family as commonwealth could be used as buffers against stressful transitions [75,76], including allowing a member to use the land as collateral security for loans. In this situation however, it is necessary to negotiate the expectations and needs of each family member [77,78].

Given the divided position on the implications of land tenure registration programmes in the literature generally, this paper seeks to present insights from the Dagbon social system in Ghana in order to explicate the situation on the ground. This follows from the realisation that inconsistencies have been common in the transformation literature [79,80], with implications for how programmes for local land registration are effective in different social structures. In this regard, analysing how actors within different social systems are involved in land tenure registration programmes and the implications of their involvement may be helpful. The core focus of this paper is therefore to explore the nature and dynamics of urban property markets in the Dagbon area in northern Ghana, and how they fare in using various land-based financing instruments. Specifically, we focus on the capacity of landed property to provide access to credit and finance investment in the housing scheme.

## 4 Materials and methods

### 4.1 Study area description

**Fig. 1:**
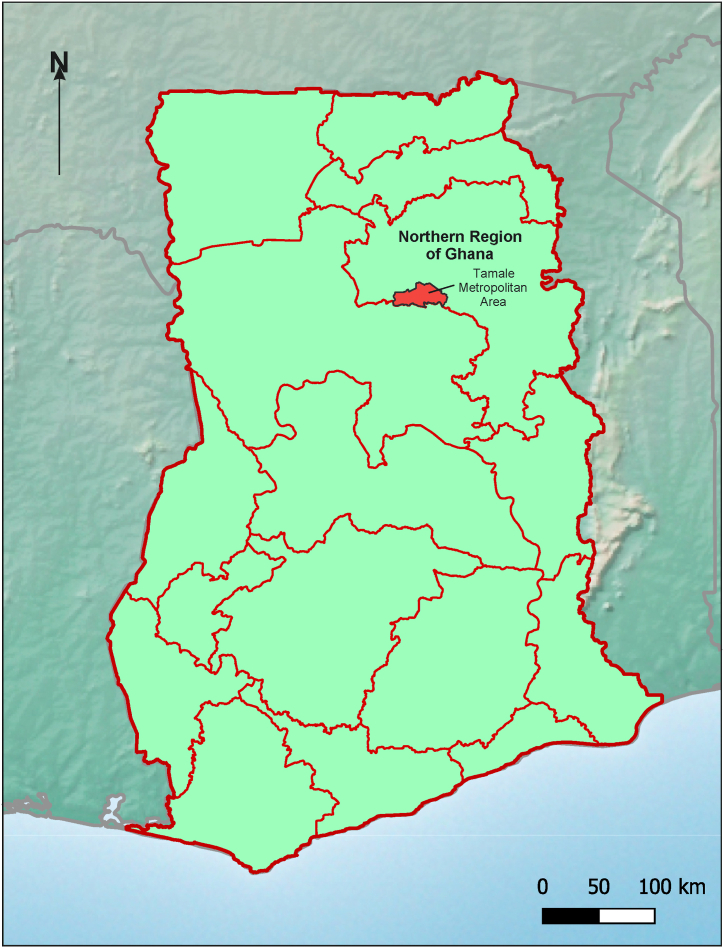
Map of Ghana showing TMA and the geographical scope of the research area.

The research was conducted in the Tamale Metropolitan Area (TMA) (see Fig. 1). Tamale is the regional capital of the Northern Region of Ghana. The indigenous people of Tamale are the Dagomba ethnic group which for centuries formed one of the oldest kingdoms in the region called Dagbon, with its traditional Overlord in Yendi. Tamale, the principal city of the Dagombas, is the third-largest city in Ghana and an evolving hotspot for investment in West Africa. Tamale serves as the administrative and commercial hub for the Northern Region but also doubles as the large metropolitan, economic, cultural, political, and financial capital of the Northern Region (see Fig. 1). The city centre of TMA hosts several local, regional, and international finance institutions and numerous international and local nongovernmental organisations. Dagbani is the indigenous language of Dagbon.

The choice of Tamale Metropolis for this study was based on the rare combination of two principal factors that make this city unique and very suitable as a research community for our purposes. In the first place, Tamale is the largest city in northern Ghana, the second most rapidly urbanizing urban centre in the country and also the third largest city in Ghana [81]. The city of Tamale as a hub of formal and business activities, is home to a number of financial institutions. Tamale has seen rapid social change with migrants from all over the northern zone, including international migrants from neighbouring Muslim countries. The records [82] suggest that by 1984 the built-up area of Tamale covered about 380 ha, which had almost tripled by 1999 (959 ha) and was 1492 ha by 2005 [83]. The built-up area of the city of Tamale has since grown to 2982 ha in 2014 with annual growth of 100 ha between 2001 and 2014. Tamale city shows unprecedented growth by expanding by 78% spatially between 2001 and 2014, at an annual rate of 4.4% [81].

Closely related to the unprecedented growth of this city is the challenge of meeting housing demands. Describing the housing situation in the Tamale Metropolis [81], observe that private individuals have become the most significant housing providers in Tamale. However, even though “… factors such as low capacity of housing providers …, high levels of poverty in the metropolis, and the sheer demand by people seeking accommodation to the point of near desperation” meant that many of the houses are poorly built with insufficient services. This trend is however quickly changing, with quality residential development emerging in the sprawling suburbs of the city [81,84,85]. [84] therefore argues that the collaborative and consultative approach to governance is having positive affects in Tamale Metropolis, especially as regards empowering the citizenry in the development of capital infrastructure.

Such empowerment includes the emergence of vibrant dynamics in urban property markets that have implications for financing instruments related to land and housing as assets. Tamale, as a Regional Capital, has a functional land title registration office where the government's coercive legal and penal force to deliberately direct policy towards land as an asset is enforced. Tamale is therefore a centre with various financial institutions that are supposed to contribute meaningfully within the policy framework to provide the support necessary for development, including housing financialisation.

The second reason for the choice of Tamale as the study community is that the Traditional Dagbon Area (Tamale being the main city of Dagbon) offers a combination of unique opportunities as a traditional system that still holds-sway in the modern era as far as land matters are concerned. Apart from the modern or central government's administrative control, the Overlord of Dagbon remains the central stakeholder in land matters. Dagbon, as the most predominantly Muslim social environment in Ghana (with polygyny as a common phenomenon across all social classes), also offers the opportunity to explore negotiations over the use of cooperative property (common-family-asset or commonwealth) as collateral to support individuals for bank loans within the small family network (be they members of the various uterine-families within a polygynous family or members of a nuclear family). Below is a map of Ghana showing the TMA.

### 4.2 Methodology

The research strategy involved the use of qualitative research (QR) [[86], [87], [88]]. In this study, the main research method associated with qualitative research were qualitative interviews used in gathering data [[86], [87], [88]]. This QR included the use of both primary and secondary data collection methods to gather information, in order to obtain the contemporary views on the impacts of land reforms on non-conventional sources of housing finance in TMA, - the capital of the Northern Region of Ghana.

For the purposes of this study, semi-structured interviews were used to elicit data from holders of landed property. These semi-structured interviews were based on a semi-structured interview guide developed by the interviewer. The semi-structured interview method was chosen for this research because this form of open-ended interview offers the opportunity to gather an extensive and in-depth array of information on the individual or group interviewed [86].

Data were collected by interviewing the credit officers to obtain their views regarding the uses of landed property as collateral to access credit from formal financial institutions for housing development. Interview guides covering the objectives of the research were prepared and used to elicit information from the interviewees (credit officers). The interview guides, amongst other things, sought to gather information on respondents’ attitudes to collateral as a requirement for granting credit, their preferences for various forms of collateral, and whether or not land must be registered to make it acceptable as collateral. The interview was conducted in TMA in the Northern Region, Ghana.

In this research, study area residents were asked about the topics of the research following a predefined interview structure. To maintain participant anonymity alongside the integrity of the data, the names of the interviewees are not mentioned in this paper.

All interviews were carried out in English, except some of those with private property owners, some of whom were illiterate. Interviews with private property owners and stakeholders who could not speak English were conducted in the local language, with the support of a research assistant (a local enumerator with not less than a decade of experience in research activities and interviews). To capture the interview data more effectively, the interviews were recorded and later transcribed by the research assistant, who is a native speaker and familiar with the language. Permission was sought from the interviewees prior to conducting electronic recordings. The recorded interviews enabled the interviewer to concentrate on the interview content and the verbal prompts and thus allowed the transcriptionist to create “verbatim transcripts” of the interviews [86,89,90].

In this research, each interview took approximately 30–50 min. All interviews with credit officers were carried out in English. In making optimum use of the interview time, the interview guides played a significant role in exploring many of the responses more systematically and comprehensively, yet keeping the interview focused on the issues being researched [86].

#### 4.2.1 Sampling technique: overview of selection process of the study population

The interviews were conducted between May and June 2016 and September 2019. Key informants and interviewees for this study were chosen through “purposive” sampling for the study [88]. Purposive sampling technique was employed to access subsets of study participants, who fit a particular profile [86,91]. This sampling technique requires researchers to have prior knowledge about the purpose of their studies, in order to choose and approach eligible participants who can help answer the research questions or achieve the research objectives [86,[91], [92], [93]]. Thus, participants were chosen for this study because they possessed the qualities that the authors sought [86,[91], [92], [93]].

With the foregoing considerations in mind, the authors decided to select the interviewees from individuals and representatives of government agencies in Tamale engaged directly or indirectly in implementing the government policy on land reforms*.* The research scope meant reaching out to people with diverse experiences of issues related to collateral as a requirement for granting credit, their preferences for various forms of collateral and whether land must be registered to make it acceptable as collateral [86,90,94,95].

As indicated by Ref. [86], since a representative sample of the population at a location could be obtained from the accessible population, findings from the sample could be generalised to that population [86,88]. The procedure included handpicking of the cases (interviewees or sample elements) involved in the study sample by the research team together with officials from the Town and Country Planning unit of the Lands Commission and the secretary to the Paramount/Traditional Chief/Council of TMA, based on the research team's judgement of the respondents' typicality [86,88]. This was a standard-based choice, which assumed that the research team aimed at finding, understanding, and acquiring further knowledge, and subsequently chose a sample in view of the qualifications and characteristics possessed by the interviewees.

#### 4.2.2 Summary of the characteristics of respondents

The research scope meant reaching out to people with diverse experiences of issues related to collateral as a requirement for granting credit, their preferences for various forms of collateral, and whether land must be registered to make it acceptable as collateral. The interviewees formed a cross-section of the metropolis (see Table 1). A total of 30 participants including 14 Credit Officers from the main Universal Banks (UBs); 2 high-ranking civil servants of the Bank of Ghana, Regional Office, Tamale; 2 Real Estate Developers in TMA, Tamale and 12 private property owners (Titleholders/Owners of landed property) (see Table 1) were selected for interviews using the following criteria:

**Table 1:** The breakdown of people interviewed in the survey and their titles/roles.

Interviewee groups/Institution	Position of interviewee	Location	No. of interviewees
Universal Banks	Credit Officer	TMA, Tamale	14
Bank of Ghana Regional Office	High-ranking Civil Servant (Head of collateral registry and financial markets)	TMA, Tamale	2
Real Estate Developers	Property developer and a Valuer	TMA, Tamale	2
Titleholders/Owners of landed property	Titleholders/Owners of landed property	TMA, Tamale	12
**Total number of interviewees**			**30**

As mentioned earlier, the research participants were selected from individuals and representatives of government agencies in Tamale engaged directly or indirectly in implementing the government policy on land reforms. Stakeholders like the chiefs of TMA, financial institutions; the credit officers from Universal Banks that provide loans, and representatives of different institutions responsible for land issues within the TMA in the Northern Region were also interviewed. Informants were also selected from the Regional Office of the Bank of Ghana in Tamale. This office represents the central monetary authority supervising and regulating financial institutions in line with the monetary policies of the Government of Ghana [96].

Representatives of the Executives of the Real Estate Developers’ Association were also interviewed. The inclusion of Real Estate Developers as interviewees provided an opportunity to gain information on real estate development processes in relation to the effect of land reforms on the impacts of non-conventional sources of housing finance in TMA. It revealed the setting in which the real estate development industry operates in TMA. Titleholders/Owners of landed property and Real Estate Developers were selected as interviewees because of their familiarity with the demand patterns for housing and housing finance in Tamale. Selected interviewees were familiar with the use of land titles as collateral to access formal credit from financial institutions for housing development.

#### 4.2.3 Secondary data collection

Secondary data were collected through the analysis of various documents relevant to the study. These documents offer important additional data on the phenomenon under study [97]. These data included institutional reports, records and papers related to the importance of Real Estate finance to developers, particularly in the Global South, and sources of finance available to private Real Estate developers in Ghana. These identified sources of housing finance then formed the basis of an analysis of the modes of accessibility and constraints of capital flow to private property owners for housing development.

### 4.3 Data analysis

Data was analysed using the method of thematic content analysis in view of its ability to describe the content of the primary data, collected by categorising common patterns and themes in the text being analysed [98,99] The process of data analysis began immediately after the group sessions, by replaying the recordings of the interviews. The data collected during the interview was carefully written down. Since an interview guide was utilised, in the case of credit officers, questions were outlined around specific ideas drawn from the literature. During data analysis, the interview transcripts, field notes, and relevant literature were analysed based on common expressions, or statements and organised themes of interrelationships among responses that addressed the aims of the study [86,100]. For each interview item, common themes across interviewees (cases) were then identified, analysed, and interpreted item by item. In this research, themes were described as patterns cutting across data sets. Themes played significant roles in the description of a phenomenon and were related to a certain research question [98,99]. This procedure enabled the researchers to make comparisons between each respondent's interviews [101]. The information gained from the interviews were transcribed [100,101] and placed into three categories in attempts to answer the research questions, as follows.

• Preference of credit officers in the study area for various forms of collateral,
• Study area residents' attitudes to collateral as a requirement for granting credit, and
• Prospects and limitations of intrafamily landed property as collateral

The method of data analysis is consistent with the purpose and approach of the study by Refs. [86,90], who suggest classifying excerpts from the transcripts into categories and identifying patterns and connections or themes within qualitative data. Validity formed an important part of this research design process, as it helps to establish the domain to which a study's findings can be generalised. External validity was ensured in this research by including theory development as a complement to the research design process of semi-structured interviews. Multiple interviewees were used to better avoid researcher bias in the study [102].

### 4.4 Limitations of the study

The study is limited to the accessible population, including stakeholders and representatives of institutions dealing with land as well as the financial institutions that provide loans for housing in TMA. These interviewees have been selected to obtain a deeper and broader understanding of the problem in the study area (TMA).

Regarding the case study design, there are certain limitations; in adopting this approach, the external validity is called into doubt because one case or a small number of cases cannot accurately represent a certain group of institutions or allow findings to be generalised to other cases or larger populations [86,103]. In this study, however, the goal is to establish a framework for discussion of the topic by concentrating on the instance of TMA and its unique setting.

In this research, insufficient sample size for statistical measurement is an issue in terms of generalising findings across social settings (i.e., external validity).

Due to the fact that neither the interviewer's nor the respondents' mother tongue is English, misunderstandings and misinterpretations of words might be a problem in this study. However, to minimise bias and enhance the reliability of the responses, all interviews were recorded. The transcripts were then forwarded to the respondents, who provided feedback on the remarks, and the material was then authorised by the interviewees [103]. Appropriate individuals who held key positions within the institutions were interviewed, reducing the possibility of misrepresentations or inaccuracies due to ignorance, and as expected, increasing the accuracy of the responses. The interviewees selected are considered the most appropriate contributors to finding answers to the research questions.

## 5 Results

As part of this research, a survey was conducted to examine the perceptions of people in the study area about the relative importance of landed property as collateral to access credit for housing development in northern Ghana. This section presents the results from interviews undertaken to gather contemporary views of the uses of land reforms on the impacts of non-conventional sources of housing finance.

### 5.1 Access to credit from financial institutions with landed property as collateral

The following section presents borrowers (titleholders & real estate developers) views on the use of landed property as collateral, as well as lenders’ (credit officers & bank managers) assessments of landed property as collateral and other critical considerations for securing a loan.

#### 5.1.1 Credit officers in study area preferences for various forms of collateral

Credit officers from the banks, title holders and real estate developers were requested to give their opinions about the use of landed property to access credit from the financial institutions. The study shows that awareness about the policy was absolute. Respondents distinguished between the use of movable properties and of landed property as collateral. Many study area respondents pointed to a caveat in the prospects of Banks accepting movable properties like vehicles as collateral. One credit officer observed:

Much as the policy has good intentions, implementing this in our society has huge challenges. Some Banks and other lending institutions have learnt some bitter lessons of how lenders could abuse this opportunity. Some customers clandestinely transfer movable properties to different locations to outwit the system.

As expected, the essence of any ownership-based system hinges on the possibility of monetizing the property of the debtor, and the legal arrangements put in place to recover assets from insolvent debtors. As noted by the respondent, however, legal arrangements to recover assets from insolvent debtors could meet with some challenges. In some cases, “… *using movable properties like vehicles …”* as collateral has been found to heighten the exposure of banks to credit risk and interest rate risks because “s*ome customers clandestinely transfer movable properties to different locations.”* In the face of such fraudulent strategies, the legal arrangements to recover assets from insolvent debtors become problematic and Banks tend to suffer losses.

The credit officer therefore thinks that Banks tend to feel safer preferring landed property with title as collateral:

Banks are currently shying away from using movable properties like vehicles, to landed properties, which are seen as much more reliable in recovery of claims.

Given the explanation, in this loan collateral or assets-based credit-accessibility system, landed property ideally meets the requirement of a property that enables the recovery of claims or effectively functions as a buffer for unforeseen insolvent debts. Findings suggest that the policy is generally seen as a realistic support for private property owners with titled landed property in particular to have the credit to invest in housing development. Real estate developers are of the opinion that another advantage of using titled landed property as collateral is the fact that, unlike other possessions, land is peculiar in its ever-appreciating value with time.

In the case of land, it is expected that the value of land in Ghana usually appreciates vis-a-vis other depreciable assets that may suffer impairment loss in the future. Landed property is further consolidated by its immovability and titling. Land titling gives confidence to prospective purchasers to develop the land without any legal problems, and generally it makes the transaction easier. Titling communicates the true ownership of the parcel of land, size, the situate and the particulars that may help to estimate its worth. Like any other capital, it shows the very particulars necessary for its presentation as a marketable commodity on the ground which can be costed. Litigation is therefore reduced to the barest minimum.

The opinion of the credit officer makes a clear distinction between titled landed property and other movable properties used as collateral, and banks preference for the former. The argument points to the significance of titled landed property, socially considered as a high-quality asset, having comparatively greater potential to be monetised or liquidated effectively.

On the other hand, however, respondents (credit officers) generally had misgivings where movable properties, by policy, could also be used as collateral to access credit from financial institutions. Almost all our respondents could cite at least two cases in which banks accepting this had been defrauded. One credit-officer noted:

The experience over the years is that unscrupulous customers could easily defraud banks using movable properties as collateral. Some tend to move say vehicles that have been used as collateral to neighbouring countries for sale. The fellow then absconds from the country for many years and not even the guarantors can easily be traced.

Accepting movable properties as collateral is generally seen by respondents as a huge challenge in Ghana, as financial institutions tend to categorise them as volatile enterprises that expose banks to comparatively high credit risk and outrageous transaction costs in tracing fraudulent debtors.

On the other hand, because land is fixed and immobile by its very nature and the documentation can be transferred legally to the lender in case of default, it is generally regarded as ideal collateral in Ghana. Respondents were, however, quick to note that, for most banks, land titling rights per se is not considered as enough to improve access to credit. Many more contextual details are necessary in the decision-making around land and credit markets in northern Ghana. One credit officer explained:

Title to land may produce the acceptable sign of genuine ownership. However, in practice, it may not wholly encompass the requisites in granting and/or approving loans. The banks usually have other considerations like: how marketable is the property in order to cover the loan obligation if need be? The transaction costs should be accessible to the borrower; and it is best when the said property constitutes a significant loss to the borrower when taken. It is even more viable when a private property is situated on the said land.

#### 5.1.2 Study area residents’ attitude to collateral as a requirement for granting credit

The basic consideration for the credit transaction of the bank is the credit worthiness of the debtor. From the perspective of this respondent, land title as collateral is granted when the lender is assured of recuperating, by court action, if need be, the value established in a given transaction. Landed property is therefore an assurance of having a property to fall back on when the borrower defaults in payment. In this wise, the respondent gives a clear indication of the fears and uncertainties associated with the transaction. Banks consider the fact that the saleability of the property may not always be as smooth as anticipated and that more latent costs may be incurred in the process. What is of paramount importance to the lender is the issue of appropriability: whether the said land can be sold readily. To our respondents, these were all critical considerations for securing a loan. In practice, the assumption that land title will bring more immediate access to credit may not be wholly tenable.

However, aside from the issue of using registered title to land as collateral, the respondents generally thought that land title registration provided an advantage in improving the value of landed property. One real estate developer observed:

Land title acquisition makes even marketing and transferring landed property easier. Previously, people wanted evidence like block fencing of a piece of land to raise the price because of the added sense of security it brought. Today, people just need documentation as security and are willing to pay more for such properties than those without. It is also partly due to the fact that changing title ownership is not as involved as when one has to engage all those land officials for making first-time documentation and titling. Titled property is much more expensive because of the sense of security. Without even physically visiting the site, those abroad who offer to purchase the titled land can be aided using satellite imagery and aerial photography to see what is on the ground as portrayed by the document.

In the opinion of the respondent, title registration increases tenure security and tends to improve the value of the land, making it easier to transfer the title to more dynamic land users. It is also interesting that, for this respondent, title to land not only facilitates easier transfers but does this through a clear indication of ownership, the size and the precise particulars. These are especially important for buyers in the diaspora who cannot physically be present but could use satellite imagery to follow events and any development activities they so demand on the land. Documentation therefore feeds into the objective expectations of all including the one paying for the land while living abroad, the family acting as the middleman and the title holder disposing of his land.

Respondents were also asked about what they perceived as major challenges in accessing bank credit using landed property. One landed property holder explains the frustrations he had to go through:

My challenge was the bureaucratic system and demands of the banks which tend to prolong the process. Apart from this, banks do not see the landed property title collateral as the single most critical decisive factor in granting loans. Other credentials come to play in the decision-making process.

The findings show an array of possibilities that come into the decision-making process of banks in giving credit. One respondent explains that landed properties are not the only security a borrower must bring for a credit facility. Other assets could include movable assets like vehicles, cash receivables etc. (depending on the nature of the requested credit facility). The bank may also consider the borrower's credit rating, cash flow statement, income, assets, or debt burden. Insured inventories may also be used when there is lien placed on the said inventory. Credit can also be accessed using investments that guarantee fixed returns, like T-bills and fixed deposits. In this case, the bank will request to have lien and set off on the investment to be used as cash back and roll over until the loan amount is fully paid.

However, the respondents expressed misgivings about the fact that the initial enthusiasm over land titling to meet promises for expanded, more inclusive access to credit was disappointing. As a result, the new generation of potential homeowners cannot get mortgages, and many businesses are struggling for loans to help them grow even when they have titled landed property as collateral. The general opinion is that banks used to regard property as a good asset for security. Currently, however, it seems to make little difference in the decision-making process. Other additional requirements are demanded that many borrowers cannot readily provide. At the practical level, aside from collateral, the bank considers other sureties including financial statements and analysis, what type of credit facility is being sought, industry analysis and how repayment is to be sourced, the type of business, and the bank's policy regarding that business. However, one respondent explained that, in practice, banks would consider the situation on a “*case-by-case basis.”* He was of the opinion that financial institutions typically offer credit to borrowers who are deemed to have terms that benefit the bank.

In this respect, the respondents noted that for some of the people seeking bank loans for estate development, the snag lay in proving that one had regular, consistent income that could pay the loan in a systematic function. Investment in private estate may not yield immediate money to service the loan on schedule. The respondents were aware that loans directly plunged into private estate development do not in themselves yield immediate returns for servicing the loan. This suggest that having registered landed property for collateral may not appeal to the lender unless, aside from the collateral, the applicant has proof of a source of incomed for a systematic repayment schedule. Thus, the loan system benefits family members with consistent sources of earnings, including salary earners and viable business individuals.

It is clear from the interviews that, for our respondents, the use of land title as collateral continues to dominate discourses in Ghana because accessing formal sector credit is more convenient for those with more stable income sources, especially those formally employed, because they have regular salaries to show and getting a title deed is about the only viable support necessary. However, the challenge is that when unforeseen circumstances arise, the bank may foreclose if there is default on the loan and then the borrower can become worse-off than before. Given the situation, a follow-up question sought to explore the opinion of respondents about what then attracts people to use land title to access credit from banks, especially when they are also very aware of the risk. One credit officer explained:

Relying on private home loan from friends and family, is very common. However, despite the comparatively better advantages including flexible payback periods and little or no interest rate, most such borrowers fail completely to pay back. Indeed, every family member here could tell you negative experiences from small monies to huge capital that never got redeemed. Relatives being relatives will not just pay back and that brings bitterness into the family. Even if you pay back, there is always a social side of that ‘favour’ that never wanes, and you and your children are supposed to be ‘indebted’ to your benefactor all your life. But the formal bank transaction is professional. Family members may allow the use of family property because apart from the family putting pressure, the banks know how to get their money back. The best the nuclear family may offer is to allow the member to use family asset as collateral with the faith that the banks will ensure repayment of the loan and the return of the asset.

Much as intrafamily mortgage is a common phenomenon, its pitfalls are great. Family loan repayment terms are usually lower than the bank's or, in most cases, usually no interest is charged at all. However as noted by the respondent, experience has shown that enforcing pay-back of the loan could be a major challenge. The banks on the other hand, are ‘professional’ and objective lenders that have the capacity to exert the necessary pressure for payback. The nuclear family asset given in support as collateral for a loan from the bank registers the family's confidence in the ability of banks to engender prompt repayment of such loans and the return of the family asset.

#### 5.1.3 Prospects and limitations of intrafamily landed property as collateral

Regarding the nature of land title as collateral, some respondents noted that, in practice, only the affluent actually possess their own individually acquired land titles because they are able to pay their way through the dense bureaucracy.

The ordinary person may have a piece of land he wants to develop, but such land is a relatively small parcel in value that the bank may not see as good collateral. However, in some families, financial capital tends to be obtained through the use of shared titled nuclear family land assets which are usually huge idle lands or family property bequeathed through the generations. Using this common family asset as collateral for the individual is usually a tough decision because of the social significance of this property and the fact that credit risk in pledging collateral to a formal lender could mean the loss of that property.

However, an interesting finding in this study, as noted by one respondent, is that despite the known high risk associated with using family property, in some families, there actually exists duly registered common family assets especially landed property, that have been serving as revolving collateral support to members. The respondent explained why this approach is feasible:

Land is one asset that appreciates in value especially with age /time and holds a good promise. Families with assets like bare land just sitting at the periphery of the city have very good economic prospects. With time, development absorbs such land with huge promises. Registering the land increases the chances of bank approval as collateral. Even when the land stands idle, and there are many like that in very good placements/locations, it could serve as a central asset for collateral support to those family members who need bank loans. The better the prospects the property offers, the more legitimacy elder siblings wield in exerting authority over decision-making regarding how the asset becomes beneficial to all. The head of family may swear affidavits and transfer the said property into the name of the applicant of the loan and that settles the matter with the bank.

In addition, with regards to the procedure for using family landed property as collateral for supporting an individual's application for credit from the bank, one credit officer explains as follows:

If it’s a family land and they have a family head/eldest sibling, he can sign the consent on behalf of the entire family at the bank. However, in instances where there is no family head, each nuclear family member will have to sign the consent form before the land can be used by the applicant of the loan.

Given the situation, a follow-up question sought to explore the opinions of credit officers on the challenges of accepting family landed property as collateral. One credit officer observed:

Sometimes when the family head signs the consent and there is a default on the credit facility, some of the family members try to prevent the bank from selling the property. As a result, banks are a little sceptical about accepting family lands as collateral for loan application. Also, it sometimes becomes difficult for banks to get buyers for a property that people know it belongs to a family, either because of the consent issues or the fact that the land belongs to a known local traditional family. Consequently, the prospective buyer wouldn’t want to be involved in such a transaction.

The family land lying idle may draw the collective attention of all members and could serve as the commonwealth and therefore one centripetal force tethering members to the family's orbit and legitimising the authority structure of family elders even in the face of rapid social change. One landed property title holder explains how, in some cases, such family property becomes a revolving asset benefitting all sequentially.

Look …, my younger brother used our family property as collateral for the loan to build his house. When it was over, another sister is doing the same. During such situations, the family is on edge, a lot of pressure to ensure the return of the family asset. Afterall no family wants to be a laughingstock. One may not get the best credit score, but registered land gives a good start because it lowers the risk to the lender. However, defaulting on a secured loan means losing that collective property. Although people plan on paying off their loans, the reality in life is that sometimes it is a gamble. The collateral could be confiscated and potentially make your family situation worse.

In a social system where family connectedness, among other things, is based on mutual family support, such assets serving as a revolving collateral support may function as the glue that holds the family together. Using familial networks presupposes that even an idle property may fit into the assets-based credit-accessibility system if registered, and becomes an add-on to the credit score and useful for negotiating premium rates as a result of the lowered risk to the lender. Accordingly, some families even reserve particular family properties to be bundled around with the common interest in raising capital “… *My younger brother used our family property as collateral … When it was over, another sister is doing same.”* As noted, however, managing intrafamily risk requires a lot of open conversation and planning within the family.

Due to its social significance, using intrafamily property is a two-edged sword. On the one hand, it comes with prospects of uniting the family across years, geographic locations, and life circumstances. On the other hand, failure to repay the loan may mean intrafamily and intragenerational feuds. By default, therefore, the family becomes a pressure group dedicated to policing the well-being of future generations by preventing this or any successor generation from squandering collective family resources. Such a collective family watchdog over the commonwealth has the added intrinsic value of raising the stakes through creating panic over the loss if the said property is confiscated. It is this consciousness that heightens the impetus to reclaim the property through prompt repayment of the loan because the opportunity cost is loss of family wellbeing.

In the study area, control over land is still dominated by the traditional order. The respondent noted that because the chiefs control issues about land, the challenge of land thugs (land-guards) as protectors of private property is generally seen as relatively minimal. However, respondents observed that intrafamily disputes are common especially where some family members feel side-lined in decisions regarding the use of registered land titles in seeking loans.

In this study, therefore, efforts were made to discover the perceived magnitude of litigation over land-based collateral in the study area. One credit officer explained:

Sometimes in the nuclear family, individuals entrusted with the management of property on behalf of the family make unilateral decisions and appropriation of the land without due consultation with other units. Even though the procedures are well spelt out in tradition and especially for Muslims, people try to undermine the system. The situation is sometimes worsened where (in polygyny) there is always a level of rivalry between the children of cowives and where male children traditionally dominate decisions. The first wife whose children may be the oldest but have only female children would have limited control. The customary law provisions and the provisions of the modern state system may not necessarily agree on what should be done. Disagreements over the common property and its management are therefore becoming more and more complex in our family system

The respondent raises some issues regarding the use of common family landed property as collateral. In some cases, the individual asset presented as collateral to the bank may be judged to be much too low in value to secure what the person objectively needs as a loan from the bank. In this sense, the collateral presented for the given loan is not optimal in relation to the terms of credit to warrant acceptance by the bank. People therefore tend to rely on nuclear family assets especially titled land to augment their own titled property to meet the demands of the lender. While this is the reality for many at some point, defaulting in payment and the threat of the loss of the collective family asset often poses a threat to the wellbeing of the larger family.

Much as using the common property is seen as necessary, intrafamily disputes may emerge when the administration of this right is seen to be exploited for the good of only a select few.

It is also interesting that the respondent introduces the dynamics of family structure into the debate. The complex issue of polygyny and, the implications of the existence of rival uterine families, mangled with the customary gender perspectives within the family in the community, are implicated in the execution of individual member requests to use the common family property as collateral. Just as intrauterine family conflicts are common in that siblings of the same nuclear family have feuds; the respondent suggest that the issue may be aggravated in polygyny. In a polygynous family set up, protecting the interest of inter-uterine families is the most critical factor in the balance of peaceful coexistence. Any wrong judgement on the part of the family elders may draw out the bad blood. Sometimes the condition is heightened not only by the idiosyncrasies of individual family members, but by challenges of inherent conflictual co-wife rivalries that transcend the individual, engulfing inter-uterine family relations that may create schisms and thwart collective aspirations.

The respondent further links the type of family and cultural definition of rights of various genders to considerations in granting the collective asset as collateral. Elders tend to favour male requests for support using the family asset for collateral within the family network. Even within a given uterine family, female children are less likely to benefit from the use of family assets for collateral.

The risks and inherent conflicts associated with using family property as collateral when the said commonwealth might be foreclosed suggests that family members would shy away from using such assets in order to avoid feuds. In this study, therefore, efforts were made to explore whether people had the experience of loan properties being so threatened or foreclosed. Interestingly, the findings suggest that most of our respondents could cite at least one person who had defaulted on loans where the threat of foreclosing on family property was to take effect. One respondent shared his own experience on the matter:

Well, I once got into the trouble of defaulting on paying a loan that I had contracted using a family property as collateral. The situation became bad because unfortunately, towards the end of the year when I contracted the loan, I was hit unexpectedly by redundancy at work. You see how things work here? Grace to grass in a sudden. The elders and the family understood my struggle, called a family meeting and agreed to take up the mysteries of the challenge. A far junior cousin offered and redeemed the family property and good name. Because of the family relationship, it was easier for family elders to negotiate for me to pay just the debt to him later. The entire family accepted to leave the family property in his hands for 5 years until I had paid back in full.

An interesting caveat emerging from our interviews in respect to customary ways of dealing with bad debts in the family is that because foreclosing on family property is traditionally an insult to the integrity of family members as individuals and as a group, the social pressure to save face and good-will may instigate the family to save the situation at the expense of a blame-game. The individual may draw the empathy of family members when such uncertainties are classified not under negligence or failure but associated with spiritual warfare, or witchcraft. The role of family elders is critical in resolving both the spiritual challenge that caused it and the physical manifestations for settling the debt. Such a crisis usually provokes the call for heroes to emerge, no matter how young, to show valour, and the ability to defend the family name. The cry from gatekeepers for help to avoid imminent disgrace immediately conjures the spirit of boldness, selflessness, and justification for assertiveness from any individual or even a younger member, which hitherto would have been regarded as condemnable and condescending. It demonstrates the latent function of family crisis shifting the boundaries of intergenerational values, its cultural contents, and how family politics shapes new values.

In this context, gatekeepers are pressured by circumstances to remodel prevalent cultural values to frame the acceptance of help from perhaps a much younger member of the family as valorous, necessary, valid, and fitting. By this redefinition of values, that which would have been regarded as patronising is hailed and redefined in the new context as god-sent. The priority here is not who pays but who is ‘selfless’ enough to stand steadfast in ensuring that the property stays in the family. The situation then turns into an intrafamily mortgage, in which, albeit informally, the borrower would be made to establish a schedule of regular repayments. This however gives the family lender the right to hold a lien on the property and have the legal right to demand full payment on the outstanding balance if the original borrower falls behind in making payments. In the event of this failing, an arrangement by family elders could be to unreservedly offer the property and the title for use by the new owner for a stipulated number of years in lieu of the debt. After that period elapses, the said property again reverts to the family reserves. Respondents noted that this customary approach of settling the debt is also helpful to the bank because, left to its obvious trajectory, it may take an average of between 2 and 5-years to sell the collateral property to defray the loan. The customary approach readily provides quick repayment to the Bank.

However, the issue raises the question as to why family members come gallantly to the rescue not when the member was initially sourcing a mortgage (the same individual family member could have provided an intrafamily mortgage) but appear unconcerned until the crisis. As noted, this is deliberate given that such family favours are easily abused. Pushing for payment usually degenerates into a family feud. Respondents further noted that people are also generally cautious about the notion of their effort to help the other relative becoming a source of envy and being misconstrued as showing largesse to belittle other members. The same interpretation may explain why many younger people would rather avoid serving as private lenders to individual family members to finance home purchase, because they may never receive repayment. A respondent explained that when crises emerge, however, the drive of elders is to emphasise two critical intergenerational duties: the opportunity to rise to the occasion and accept the claim of a role model in the context of the duty to repair any damage caused by the failure of anyone in order to maintain the family's good name; and availing oneself as a conduit for passing the family inheritance on to generations yet unborn in good condition. The family, having taken the responsibility as a group, would have to ensure that the debt was paid, or the family property confiscated to the family lender.

## 6 Discussion

The study reveals that reforms to enhance credit access with landed property for collateral is seen by all respondents as the most critical factor that could change access to loans for real estate development finance. However, the findings suggest that for some, finding relevant collateral to support their application could still be a challenge. In some cases, the assets that individuals possess are judged to be too low in value to warrant acceptance by the bank as collateral for the amount of capital desired. People therefore tend to rely on nuclear family assets, especially titled land to augment their own titled property to meet the demands of the lender. While this is the reality for many at some point, defaulting in payment and the threat of the loss of the collective family asset often poses a threat to the wellbeing of the family. Indeed, the difficulty faced by the poor in finding befitting collateral and the possibility of foreclosure in case of default has been discussed extensively in the literature. Several studies [104,105] admit that the primary challenges underlying the management of land markets in developing countries are the complex relationships between people and land.

On the issue of the type of collateral that banks are more favourable towards, respondents pointed to immovable property, especially landed property. It is the general opinion that accessing finance with movable collateral is comparatively rare in the study area as has been found to heighten exposure of banks to credit risk and interest rate risks. This finding agrees with instances cited by Ref. [12], where, in some cases, borrowers mortgaged such movable assets to more than one financial institution. They assert that, management cost on movables on post disbursement is high, as this process must be continued until the full recovery of the advance. In their view, a large percentage of nonperforming loans is secured against movable assets.

On the other hand, however, the findings in this study suggest that there is unanimous acceptance among respondents (credit officers) that landed property ideally meets the requirement of a property which effectively functions as a buffer for unforeseen insolvency. This finding agrees with a number of other studies [106,107] that title registration increases tenure security and affords landholders a title that can be used as collateral with financial institutions. This finding is further consistent with works on title registration by a number of other studies [108,109] that have respectively argued that the absence of secure land title breeds land litigation due to incidents of encroachment and multiple land ownership [9].strongly recommends title registration as a panacea to collateral challenges for the banks in Ghana.

However, a critical look at our findings still poses questions about the fact that, despite the provision of collateral, banks demand evidence of a systematic source of cash flow, the financial statement and analysis, industry analysis, and how repayment is to be sourced. Private estate development does not yield immediate money for servicing the loan. Seeking collateral support is a non-starter for the many who are unable to prove viable repayment through regular income. The use of collateral is thus largely to the advantage of those who have proof of secure income. The bigger picture is that, using landed property as collateral runs the risk of having to forfeit the property when one is unable to pay back the loan to the bank. Opposing the assertion of [29], therefore, some of our respondents say they find it suicidal to pledge their only productive family asset in the assets-based credit-accessibility system. The literature is split on the issue. Contrary to our findings, literature also shows evidence that title registration programs have produced good results in some jurisdictions [110].

Our study further made contributions to dynamics in the role of intrafamily assets, especially titled land in augmenting the private titled property of individuals to meet the demands of the lender. Our findings suggest that family feuds are common when some family elders abuse their role as seniors to annex such properties or allot the privileges without consultation. Feder et al. [[11], [111]] observe that the land title register is always the final authority in ascertaining the validity of land documentation. The state tends to accept responsibility only for the validity of transactions that have been entered into the land title register. Since title registration offers a system of conveyance that is complete and dispenses with the need for investigation of title by persons dealing with registered land, the head of family wields a lot of discretion. Our finding therefore agrees with the work of [112], suggesting that persons entrusted with the management of lands on behalf of groups cannot make unilateral decisions concerning the appropriation of the land without consulting the principal members of the group. His work reveals how the neatly laid out de jure provisions for the management of land in Ghana, which principally recognises the head of family can present tensions in the de facto application of the laws.

The study reveals that securing loans for real estate development in the target area has been seen by all as a daunting challenge. Our findings show that titling is not sufficient to gain access to collateral-based credit for estate development in the target community. Other additional requirements including the financial statement and analysis, what type of credit facility is being sought, industry analysis and how repayment is to be sourced, the type of business and the bank's policy regarding that business. This finding confirms a number of other studies [35,37,46,47], that show the limitations of theory as applied in practice generally in developing countries**.** [113] found in Indonesia for example that land title does not, in and of itself, guarantee access to credit, but rather provides only one requirement among many regarding the borrower's creditworthiness. Likewise, a number of other studies [32,110,114] also report that the majority of their respondents believed that having secure land title alone did not improve their prospects of securing a loan using the issued land certificate as collateral.

It is within this context that the [41] assertion becomes appealing. They contend that the concept of secure tenure is complex, involving the trilateral concepts of legal, social and economic factors. They advised that it should be considered as a multidimensional concept which calls for wide strategic and policy making initiatives (ibid.: 328). In assessing the findings of this research within the rationale upheld by Ref. [41] therefore, it should be emphasised that the variables which impact lending decisions are multi-faceted and varied.

The findings of this study also agree with a number of other studies in the Sub-Saharan African region that indicate no link between land title registration and investment in land and especially investment in housing [[115], [116], [117]].

Contrary to the conclusion of [28] that traditional land tenure systems offer security and certainty in places where the traditional landholders have legal ownership and authority over their land and resources, the case of Dagbon demonstrates how the co-existence of both the traditional and modern concepts could reinforce each other for the security of land and property. Our study found that even though land titling may not directly guarantee bank credit, it does limit litigation over the land. In our research community (Dagbon Traditional Area), our findings suggest people who acquire land titles usually feel secure because of the role of the traditional council over land matters. The allodial title holder is the Overlord of Dagbon, and the strict traditional system of land management is firmly controlled by the traditional council working in concert with designated government agencies. Respondents were therefore generally confident that their tenure security would be guaranteed by their ownership of a title certificate [99,118].

Unlike the stance presented by Ref. [28], the case study of TMA shows no contradictions with the Declarations and Resolutions of the FAO/USP/RICS Foundation South Pacific Land Tenure Conflict Symposium held in Suva, Fiji from 10 to 12 April 2002. The findings from our study agree with the main Declarations and Resolutions of the FAO/USP/RICS Foundation, including: there are many stakeholders concerning land; the customary nature of land ownership and control should be appreciated; and commitment to good governance in relation to land tenure systems, incorporating wide participation of both the traditional and modern systems and ensuring equal recognition and empowerment for both systems, is critical [119].

The challenge of intrafamily disputes over land that is already certified with title certificates was noted by our respondents as becoming a more complex phenomenon in the research community. However, this challenge is usually not directly under the jurisdiction of the traditional system and is largely handled by the court system. Our findings therefore agree with the [32,120], observation that, land titling provides legal clarity about land tenure, formal records of property rights, effective contract enforcement and dispute resolution mechanisms. These are important in allowing lenders to assess and price risk, reduce transaction costs in doing a loan deal, and enforce their rights in the event of loan default.

In summary, the present study was undertaken to examine the relative significance of landed property as collateral to access formal credit from financial institutions for housing development in TMA in the Northern Region of Ghana. More specifically, the aims of the study were to investigate monogamous family units as well as uterine families of polygynous homes, which is a common phenomenon in northern Ghana. Thus, it explored, among other things, how members of Dagbon family structures use revolving titled land-based credit-accessibility instruments. This study sought to answer the principal research question: does formal documentation of landed property as collateral help in accessing formal credit for housing development in the Tamale Metropolitan Area (TMA) in the Northern Region of Ghana? The findings reveal a complex relationship between landed property and access to formal credit, where the results are sometimes in line and sometimes at odds. The findings generally indicate that, while landed property could possibly influence the decision to grant formal credit, it does not necessarily lead to approval in the final lending decision. Thus, findings show that for most banks, formalising property titles alone will not be enough to improve access to credit. The decision-making process pertaining to the land and credit markets in northern Ghana needs much more contextual information.

## 7 Conclusion

The present paper examined the relative significance of landed property as collateral to access formal credit from financial institutions for housing development in TMA in the Northern Region of Ghana.

The results from this case study indicate that reforms to enhance credit access with landed property for collateral is seen by all respondents as the most critical factor that could change access to loans for real estate development finance. As land is fixed and immobile by its very nature and documentation can be transferred legally to the lender in case of default, it is generally regarded in Ghana as ideal collateral. Respondents were, however, quick to note that, for most banks, land titling rights per se are not considered enough to improve access to credit. Many more contextual details are necessary in the decision-making around land and credit markets in northern Ghana.

On the issue of the type of collateral that banks are more favourable towards, respondents pointed to immovable property especially landed property. On the other hand, however, the findings in this study reveal unanimous acceptance among respondents (credit officers) that landed property ideally meets the requirement of a property which effectively functions as a buffer for unforeseen insolvent.

In sum, our findings agree with other studies that while formal documentation of land rights matters in the broad scheme of financial sector development or financing real estate development, it is not sufficient to bring about more immediate access to credit, especially for those who lack regular or reliable source of income that could be critical in ensuring the payback of loans contracted using titled land as collateral. Hence, it could not be a panacea for the challenges facing real estate finance in Northern Ghana.

The case study fails to find any direct relationship between titling and increased access to formal credit. Thus, titling is not sufficient to gain automatic access to collateral-based credit for estate development in the target community. Given this finding, the introduction of collateral registry and credit reference as a measure of borrowers’ creditworthiness should be vigorously implemented on a broad scale to augment access to collateral-based credit for estate development. This also raises the issue of policy formulation and programmes aimed at enhancing credit access with landed property as collateral to prioritise a sustainable system that ensures payback of loans contracted.

To further buttress the generalisation of the findings from this case study approach that there is no correlation between land titling and increased access to formal credit for housing development, further research is needed on collateral properties of land by either employing quantitative research methodology in data collection and analysis with a large sample size or mixed methods in the case of the same research site. Also, the authors suggest that similar studies should be carried out by researchers on a multiple-case study design in other parts of Ghana because of the diversity in cultural systems of local land management. This would open avenues for researchers to compare the data from different cases as well as consider what is common and what is distinctive across the cases using a multiple-case study design.

## Author contributions

David Asante Edwin initiated the idea of the study; David Asante Edwin, Evam Kofi Glover and Edinam K. Glover were involved in designing the research methodology; David Asante Edwin took the lead in collecting the data, analyzing, drafting the paper including discussions; Evam Kofi Glover supervised the fieldwork and contributed to the interpretation of results. Edinam K. Glover verified the analytical method and all authors provided invaluable comments that helped shape the research, analysis, and manuscript. All authors have read and agreed to the published version of the manuscript.

## Funding

This research received no external funding.

## Informed consent statement

Informed consent was obtained from all study participants.

## Declaration of competing interest

The authors declare that they have no known competing financial interests or personal relationships that could have appeared to influence the work reported in this paper

## Footnotes

1 Provisional National Defense Council Law (PNDCL).
